# Supplementary material for: Associations between damage location and five main body region injuries of MAIS 3–6 injured occupants
Source: BMJ Open. 2014 May 8;4(5):e004371. doi: 10.1136/bmjopen-2013-004371 (PMC4024600; doi:10.1136/bmjopen-2013-004371)
Supplement: Author's manuscript [file bmjopen-2013-004371.draft_revisions.pdf]

Associations between damage location and five main body regions injuries of MAIS 3-6 injured occupants

Journal:	*BMJ Open*
Manuscript ID:	bmjopen-2013-004371
Article Type:	Research
Date Submitted by the Author:	31-Oct-2013
Complete List of Authors:	tang, youming; Xiamen University of Technology, School of Mechanical and Automotive Engineering; Hunan University, State Key Laboratory of Advanced Design and Manufacture of Vehicle Body<br>Cao, Libo; Hunan University, State Key Laboratory of Advanced Design and Manufacture of Vehicle Body<br>kan, steven; The George Washington University, National Crash Analysis Center
**Primary Subject Heading**:	Public health
Secondary Subject Heading:	Public health
Keywords:	PUBLIC HEALTH, Health & safety < HEALTH SERVICES ADMINISTRATION & MANAGEMENT, ACCIDENT & EMERGENCY MEDICINE

SCHOLARONE™
Manuscripts

For peer review only

Associations between damage location and five main body regions injuries of MAIS 3-6 injured occupants

Youming Tang,[1, 2, 3] Libo Cao,[2] Steven Kan[3]

[1] School of Mechanical and Automotive Engineering, Xiamen University of Technology, Xiamen, Fujian, China

[2]State Key Laboratory of Advanced Design and Manufacture for Vehicle Body, Hunan University, Changsha, Hunan, China

[3]National Crash Analysis Centre, The George Washington University, Ashburn, Virginia, USA

Correspondence to Youming Tang, School of Mechanical and Automotive Engineering, Xiamen University of Technology, Xiamen, Fujian, 361024, China; tangyouming@xmut.edu.cn or tangyouming.academic@gmail.com

For peer review only

ABSTRACT

Background Since 1980, there has been an increase in fatal injuries resulted from the crashes of passenger cars (PC) and light truck vehicles (LTV).

Objectives To estimate the overall maximum Abbreviated Injury Score (MAIS) 3-6 injured occupants of struck vehicle which were associated with specific location of damage in front-to-side impact crashes.

Methods Data were obtained from the US-National Automotive Sampling System/ Crashworthiness Data System (NASS/CDS) in the year 2007. The five main seriously injured body regions for MAIS 3-6 injured occupants were head/face/neck, chest, pelvis, upper extremity and lower extremity. Struck vehicle collision damage was classified in a three-dimensional system according to J224 Collision Deformation Classification of SAE Surface Vehicle Standard.

Results The lateral crush zone contributed to MAIS 3-6 injured occupants and 50% injury risks when extended into zone 3 (crush intrusion between 40 centimeters and 60 centimeters). When the crush extended to 80 centimeters, the injury risk ratio of MAIS 3-6 injured occupants was approached to 85%. The horizontal crush zone contributed to highest injury risk ratio of MAIS 3-6 occupants were zone “D” and zone “Y”, and the injury risk ratio were 25.5% and 29.9%, respectively. In contrast, the lowest injury risk ratio was 3.75% caused by zone “B”. The vertical crush zone contributed to highest injury risk ratio of MAIS 3-6 occupants was zone “E”, the injury risk ratio was 64.8%. In contrast, the lowest injury risk ratio was 0.09% caused by zone “G+M”.

Conclusions The highest injury risk ratio of MAIS 3-6 injured occupants caused by crush intrusion between 40 centimeters and 60 centimeters in a LTV-PC side impact, and the damage region of struck vehicle were the zone “E” and “Y”.

ARTICLE SUMMARY

Strengths and limitations of this study

- Gain a better understanding of stuck vehicle characteristics and crash conditions in the current and future side crash environment.
- The 50th percentile risk of MAIS 3-6 injuries to a front-to-side occupants between LTV and PC correspond to extent of damage in the zone 3, specific horizontal location of damage in the zone “Y”, and specific vertical location of damage in the zone “E”.
- To reduce the risk of deaths and fatal injuries of MAIS 3-6 injured occupants, considerate head, chest and pelvis injury prevention priority.
- The injury risk ratio of five main body regions of MAIS 0-2 injured occupants were excluded.

INTRODUCTION

Since 1980, there has been an increase in fatalities resulted from the collision of passenger cars (PC) and light truck vehicles (LTV), because LTV have became the majority of vehicles on the road, resulting in great occurrences of vehicle incompatibility between the body structures of LTV and PC.[1-4] LTV have the same truck frames include sport utility vehicles (SUV), pickup trucks, and vans weighted no more than 2041kg.[5]

The reinforcement of PC is most commonly a single beam at the mid-door or lower door cage, leaving the upper portion of door unprotected. When LTV collide with PC, the higher bumper frame contacts the door above the reinforcement, creating upper thoracic and head injuries.[6-7] Hence, vehicle mismatch between PC and LTV is associated with deaths and seriously injuries of occupants in automotive crashes.[8] In the present study, US national database was used to assess the side impact injury risks of five main body regions of maximum Abbreviated Injury Score (MAIS) 3-6 injured occupants between PC and LTV.

MATERIAL AND METHOD

Database of national automotive sampling system/ crashworthiness data system

The objective was to estimate the injury risks of MAIS 3-6 injured occupants by extent of damage for impacted vehicle in front-to-side impact crashes used real-world crash cases extracted from database of National Automotive Sampling System/ Crashworthiness Data System (NASS/CDS). The NASS/CDS is a detailed representative sample of motor vehicle crashes that occur within the United States. Each year roughly 5,000 detailed crash investigations are conducted by NASS

For peer review only

investigators. Each case in the sample is assigned a weighting factor, which is equivalent to the inverse probability of such a vehicle incident occurring in a given year. Crashes occurred in the year 2007 were selected; the number of crash cases is 4,963 and after weighted is 2,454,014 collected by National Highway Traffic Safety Administration (NHTSA).

Selection criteria

In order to extract relevant data from the NASS/CDS datasets, the following selection criteria were established.

- Cases which only two vehicles were involved in the collision.
- Cases that resulted in a vehicle rollover were excluded.
- Crash configuration involved a front-to-side impact.
- Both of the vehicles involved could be classified as passenger car, and light truck vehicle including mini-van, sport utility vehicle, van, and light pickup truck.
- Striking vehicle was defined as an impact which resulted in the primary general area of damage being located at the front of the vehicle, also defined as having a direction of force between 330^0 and 30^0 (11'o clock to 1'o clock).
- Struck vehicle was defined as an impact which resulted in the primary general area of damage being located on either the right or the left side of the vehicle, also defined as having either a direction of force between 30^0 and 150^0 (1'o clock to 5'o clock right side impact), or between 210^0 and 330^0 (11'o clock to 7'o clock left side impact).

Collision deformation classification

According to the "J224 Collision Deformation Classification" of SAE surface vehicle standard, the extent of residual deformation induced by a vehicle was represented from horizontal, lateral and vertical direction.[9] For initially vehicle, the extent of lateral damage is classified using a nine-zone extent system relative to specific points on the vehicle structure. The extent of damage recorded, is dependent on the maximum cross-section to which the deformation extends to within a vehicle as a result of an impact. This measure also can be used to indicate the vehicle severity of an impact.

Vehicle horizontal areas to be used in locating the deformation for three independent extents and three composite extents, such as zone "P", "F", "B" and "Y", "Z", "D", respectively. "F" and "B" are side deformation areas forward and rearward of "P", respectively.

The classifications for the vertical location of deformations associated with impacts classified as "A", "E", "G", "M", or "L". The extents zone "A" and "E" are used for vehicle with both side deformation located rear-end, while "G", "M", and "L" located front-end. Zone "A" is used to classify impacts where the vehicle deformation resulted from an overhanging structure shaped like an inverted step in which the vertical surfaces are at least 760 millimeters apart.[9]

RESULTS

PC-LTV crashes

The information presented in table 1 show that about 55% of road vehicle crashes resulted from vehicle-to-vehicle impact. These crashes also caused about 62% of the injuries to the occupants. As it can be seen, vehicle-to-vehicle crashes result in more seriously injuries per collision than single-vehicle crashes and multiple-vehicle crashes.

Table 2 shows the injury risks ratio of MAIS 3-6 injured occupants involved in two vehicle front-to-side crashes. The highest risk ratio of MAIS 3-6 injuries was 1 versus 2.09, which resulted from PC-to-PC side impact crashes. Comparatively the LTV-to-LTV side impact crashes caused the lowest injury risk ratio for MAIS 3-6 injured occupants.

Table 1 Investigation of road vehicle crash types in US in 2007

crash type	weighted-cases	weighted-occupants (not including pedestrians)
single-vehicle	832,385 （33.9%）	1,198,553 （19.9%）
vehicle-to-vehicle	1,361,888 （55.5%）	3,753,285 （62.2%）
multiple-vehicle	259,741 （10.6%）	1,082,825 （17.9%）
total	2,454,014 （100%）	6,034,663 （100%）

Table 2 MAIS 3-6 injured occupants involved in two passenger vehicle front-to-side crashes

For peer review only

crash configuration	injury risk ratio of MAIS 3-6 injured occupants (striking vehicle/struck vehicle)
PC-to-PC	1 (2,088): 2.09 (4,354)
PC-to-LTV	2.06 (667): 1 (324)
LTV-to-PC	1 (1,573): 1.84 (2,897)
LTV-to-LTV	1 (1,398): 1.49 (2,081)
total	1 (5,726): 1.69 (9,656)

Specific lateral location of damage

Approximately 39.5% of MAIS 3-6 injuries were covered the extent zone 3 of damage. Zone 2 and zone 4 were comprised of only 19.9% and 23% of MAIS 3-6 injuries (table 3). As it can be seen, the crush zone attributed to occupants serious injured were zone 2, zone 3 and zone 4. 50th percentile of MAIS 3-6 injuries occurred when extent of damage crush into zone 3 of the struck vehicle.

The information in table 1 shows that the majority of AIS 3-6 injuries for head/face/neck (37.68%), chest (33.64%), pelvis (45.5%), upper extremity (43.62%) and lower extremity (42.69%) were associated with an extent of damage in zone 3. The extent of damage in zones 2 and 3 accounted for about 56.2% of head/face/neck injuries, about 55.65% of chest injuries, about 72.73% of pelvis injuries, about 62.88% of upper extremity injuries and about 59.45% of lower extremity injuries, respectively.

Assuming a passenger car's width was 1.8 meters in LTV-to-PC side impact crashes, the each extent of damage was 20 centimeters. Zone 1 demonstrated crush intrusion in lateral location of damage was between 0 and 20 centimeters, zone 2 demonstrated crush intrusion was between 20 centimeters and 40 centimeters... zone 9 demonstrated crush intrusion was between 160 centimeters and 180 centimeters and included broken crush, according to J224 Collision Deformation Classification of SAE Surface Vehicle Standard. However, the distance commonly between occupants and interior panel is less than 20 centimeters. Therefore, crush intrusion of vehicle side components is almost up to 60 centimeters will cause the occupants to lose capacitating or even fatal injury. In current study, the risk ratio of MAIS 3-6 injuries was 85% high resulted from the extent of damage extended into zone 3.

Table 3 Distribution of MAIS 3-6 injured occupants by body regions and lateral location of damage in US in 2007

lateral location of damage		1	2	3	4	5	6	7	8	9
occupants	Percentage	1.78%	19.87%	39.54%	23.01%	9.83%	3.97%	1.60%	0.20%	0.20%
number	Cumulative percentage	1.78%	21.65%	61.19%	84.20%	94.03%	98.00%	99.60%	99.80%	100.00%
	head/face/neck	2.92%	18.52%	37.68%	25.66%	10.72%	1.62%	2.27%	0.31%	0.31%
five main	chest	1.28%	22.01%	33.64%	21.15%	14.67%	5.61%	0.82%	0.41%	0.41%
body	pelvis	1.00%	27.23%	45.50%	18.92%	3.15%	1.50%	1.50%	0.75%	0.45%
regions	upper extremity	1.90%	19.26%	43.62%	24.36%	6.44%	3.19%	0.61%	0.31%	0.31%
	lower extremity	1.02%	16.76%	42.69%	21.32%	7.63%	7.09%	2.53%	0.49%	0.49%

Specific horizontal location of damage

The two highest injury ratios of MAIS 3-6 injuries were 25.5% and 29.9% respectively covered the damage location "D" and "Y" (table 4); comprised the lowest injury ratio of MAIS 3-6 injuries was only 3.75% covered the damage location "B".

Over half of all body AIS 3-6 injuries were contributed to the damage location "D" and "Y". The highest risk ratio of MAIS 3-6 injuries for head/face/neck, chest, pelvis, upper extremity and lower extremity was respectively about 28.5%, 33.5%, 22.6%, 32% and 35.5% associated with the damage zone "Y". In contrast, the lowest risk ratio of MAIS 3-6 injuries for head/face/neck, chest, pelvis, upper extremity and lower extremity was only about 5%, 2.7%, 6%, 2.7% and 3.2% respectively associated with the damage zone "B".

For peer review only

Table 4 Distribution of MAIS 3-6 injured occupants by body regions and horizontal location of damage in US in 2007

horizontal location of damage		unknown	B	D	F	P	Y	Z
occupants number	Percentage	0.43%	3.75%	25.47%	16.35%	11.07%	29.88%	13.20%
five main body regions	head/face/neck	0.53%	5.02%	22.41%	20.87%	8.70%	28.24%	14.24%
	chest	0.66%	2.66%	30.03%	10.83%	12.14%	33.50%	10.18%
	pelvis	0.00%	6.00%	17.85%	17.85%	15.48%	22.59%	20.22%
	upper extremity	0.00%	2.74%	20.09%	15.75%	10.84%	32.08%	18.49%
	lower extremity	0.48%	3.17%	25.22%	15.76%	12.16%	35.11%	8.09%

Specific Vertical Location of Damage

Approximately 64.8% of MAIS 3-6 injuries were resulted from the damage location “E” (table 5); zone “G+M” was comprised of only 0.09% of MAIS 3-6 injuries.

As it can be seen, the majority of all MAIS 3-6 injuries were contribute to the damage location “E”, the risk ratio of MAIS 3-6 injuries for head, chest, pelvis, upper extremity and lower extremity was about 62.5%, 55.5%, 64.5%, 77% and 69%, respectively.

Table 5 Distribution of MAIS 3-6 injured occupants by body regions and vertical location of damage in US in 2007

vertical location of damage		unknown	A	E	G+M	L
occupants number	Percentage	3.32%	31.69%	64.74%	0.09%	0.17%
five main body regions	head/face/neck	4.40%	32.81%	62.52%	0.00%	0.27%
	chest	3.16%	40.83%	55.23%	0.37%	0.41%
	pelvis	4.69%	29.38%	64.55%	0.00%	1.38%
	upper extremity	1.63%	20.31%	76.93%	0.56%	0.56%
	lower extremity	2.62%	28.02%	68.74%	0.10%	0.51%

DISCUSSION

We evaluated the risks of MAIS 3-6 injuries of head/face/neck, chest, pelvis, upper extremity and lower extremity from two passenger vehicle front-to-side impact crashes. Our analysis was based upon an examination of approximately 1,362,000 weighted cases of PC and LTV which were extracted from the US-NASS/CDS 2007 crash investigations database.

Previous studies have indicated occupants of PC were more likely than occupants of LTV to be seriously injured. About 2.9% of PC occupants and 1.9% of LTV occupants in side-impacted vehicles were seriously injured.[10] Crash aggressivity index (CAI) of LTV, compared to the reference case PC (0.00), the increased from vans (0.32) until medium truck (0.48), and then for heavy truck (0.40) and truck tractor (0.21) it decreased.[11] PC-LTV incompatibility in vehicle mass, vehicle geometry and structure also have been widely discussed.[12-14]

In our study, the injuries risk ratios in front-to-side impact crashes was that overall occupants of struck vehicle have a sixty-nine percent higher MAIS 3-6 injury risks than overall occupants of striking vehicle. The reason is that the striking vehicle occupants are, to a greater extent, protected by the presence of a larger structural crush zone, i.e. including the energy-absorbing bumper and front-end structure, the vehicle’s engine, the front suspension and wheels, the engine-mounting frame and integral firewall, supplemental safety systems. However, the struck vehicle crush zone is comprised of only the side doors and the relatively light framework of the occupant cell, plus interior foams which offer some limited protection, or some vehicle types are now also equipped with supplemental side airbags or curtains which have increased the level of side impact protection available to the occupants, but the level of protection does not reach the level which currently exists for frontal crashes.

The results from lateral damage location suggest that strategies and methodologies of occupant protection and injuries reduction are developed from the extent of

For peer review only

damage in zones 1, 2 and 3. Two methods are often used to improve vehicle safety in side impact crashes. Method number one is enhancing the structural rigidity of interior panel of side doors to reduce magnitude of door panel crush, such as optimized design of impact bar, foam systems and door interior panels. Because magnitude of door crush in near-side impact from category 1 (3-8 centimeters of crush) to category 2 (8-15 centimeters of crush) is associated with an average of 0.4 increase in thoracic AIS, a 0.3 increase in abdominal AIS, and a 0.2 increase in pelvis (p<0.0001).[5] Method number two is adding the airbags between the occupant and interior panel, i.e. supplemental head curtains and chest airbags which have increased the level of protection available to occupants.

The results from horizontal damage location suggest that the primary crushed location of impacted vehicle is forward of c-pillar in real world side impact crashes, and it is less possibility to impact the rearward of c-pillar for target vehicle. Therefore, for design researches it is useful to maintain the side rigidity of vehicle specially accounted for structure rigidity of side doors, A-pillar, B-pillar and rocker rail.

The results from vertical damage location suggest that the primary crushed location of impacted vehicle is located in upward of rocker rail and downward of windowsill in real world side impact crashes, and it is less possibility to impact the upward of windowsill for target vehicle. Considering improving the level of side protection particularly should enhance the rigidity of side doors and rocker rail from perspective of vertical anti-crushed location of vehicle, or consider vehicle front end design, especially for LTV, in motor vehicle safety standards.[15]

In conclusion, over half of crash types in real world are attributed to vehicle-to-vehicle crashes. Rigidity of side doors, A-pillar, B-pillar and rocker rail are priorities for improving the safety level of side impact. Because the specific location of damage accounted for MAIS 3-6 injured occupants and 50% injury risk is extent zone 3, zone "Y" and "E" from lateral, horizontal and vertical direction of struck vehicle, respectively. To reduce the risk of deaths and serious injury, protection of head, chest and pelvis should be priorities for injury countermeasure development, because these three body regions accounted for over half of AIS 3-6 injuries suffered from vertical crush zone "E" in PC-LTV side impact crashes.

Acknowledgements We would like to thank Dr. Prodeep Mohan (senior scientist of National Crash Analysis Center, George Washington University), Ms. Christina (master graduate student of National Crash Analysis Center, George Washington University), and Mr. Leyu Wang (doctoral graduate student of National Crash Analysis Center, George Washington University) for their very valuable suggestions and software processing to this work.

Contributors YT was the primary investigator, he initiated and designed the study and completed the data processing and manuscript writing. LC and SK participated in developing the study concept and data interpretation. All authors reviewed and approved the final manuscript.

Funding This project was supported by National Crash Analysis Center (NCAC), the George Washington University (GWU) and sponsored by National Natural Science Foundation of China, grant no.51305374. Open Foundation of State Key Laboratory of Advanced Design and Manufacture for Vehicle Body of Hunan University, grant no. 31215004. Fujian Natural Science Foundation of China, grant no. 2012J05103.

Competing interests None.

REFERENCES

1. **Acierno S**, Kaufman R, Rivara FP, at al. Vehicle mismatch: injury patterns and severity. *Accid Anal and Prev* 2004; **36**: 761-772.
2. **Bradsher K**. The triumph of SUVs. *High and mighty*. 1st Ed. New York: Public Affairs, 2001:382-412
3. **Samaha RR**, Elliott DS. NHTSA side impact research: motivation for upgraded test procedures. Nagoya, Japan, May 2003. *18th Conference on the Enhanced Safety of Vehicles (ESV)* 2003; paper No.492:1-17
4. **Mandell SP**, Mack CD and Bulger EM. Moter vehicle mismatch: a national perspective. *Inj Prev* 2010; **16**: 309-314.
5. **Crashworthiness Data System** (CDS) 2007 coding and editing manual. Washington D.C: Transportation National Highway Traffic Safety Administration, 2007.
6. **Tencer AF**, Kaufman R, Mack C, et al. Factors affecting pelvic and thoracic forces in near side impact crashes: a study of US-NCAP, NASS and CIREN data. *Accid Anal and Prev* 2005; **37**:

For peer review only

287-293.

7. **Insurance Institute for Highway Safety** (IIHS). Putting the crash compatibility issue in perspective. *Insurance Institute for Highway Safety Status Rep*ort 1999; 34:1–11.
8. **Eric MO,** Thomas DK and Barbara M. Will the light truck bumper height-matching standard reduce deaths in cars?. *Traf Inj Prev* 2013; **51**: 292-300.
9. **Society of Automotive Engineering**. *Collision deformation classification of surface vehicle standard-1980*. Warrendale: SAE, 1980.
10. **Charles MF**, Elisa RB and Eric LM. Two-vehicle side impact crashes: the relationship of vehicle and crash characteristics to injury severity. *Accid Anal and Prev* 1997;**29**: 399-406.
11. **Helai H**, Chowdhury S and Mohamed AA. Indexing crash worthiness and crash aggressivity by vehicle type. *Accid Anal and Prev* 2011; **43**: 1364-1370
12. **Toy EL** and Hammitt JK. Safety impacts of suvs, minivans, and pickup trucks in two-vehicle crashes. *Risk Anal* 2003; **23**: 641-650.
13. **Kahane CJ**. Vehicle weight, fatality risk and crash compatibility of model year 1991-99 passenger cars and light trucks. *NHTSA Technical Report* No. DOT HS 809 662, 2003.
14. **Fredette M**, Mambu LS, Chouinard A, at al. Safety impacts due to the incompatibility of suvs, minivans, and pickup trucks in two-vehicle collisions. *Accid Anal and Prev* 2008; 40:1987-1995
15. **Roudsari BS**, Mork CN, Kaufman R, et al. Pedestrian crashes: higher injury severity and mortality rate for light truck vehicles compared with passenger vehicles. *Inj Prev* 2004; **10**:154-158

For peer review only

According to the "J224 Collision Deformation Classification" of SAE surface vehicle standard, the extent of residual deformation induced by a vehicle was represented from horizontal, lateral and vertical direction as shown in figure 1 to figure 3.

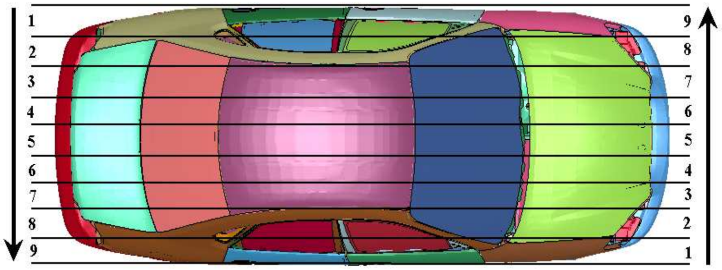

Figure 1. Specific lateral location of damage

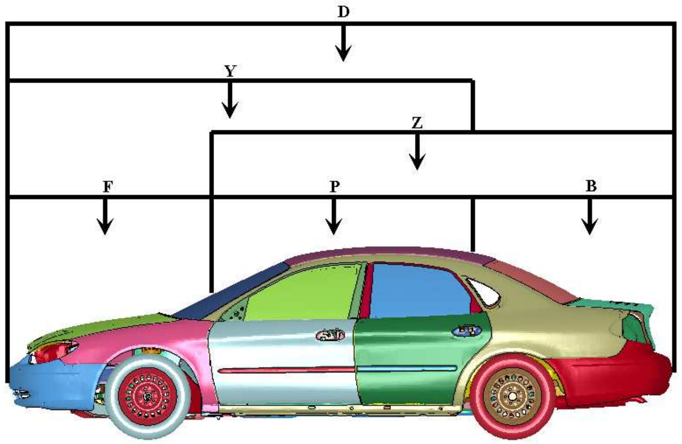

Figure 2. Specific horizontal location of damage

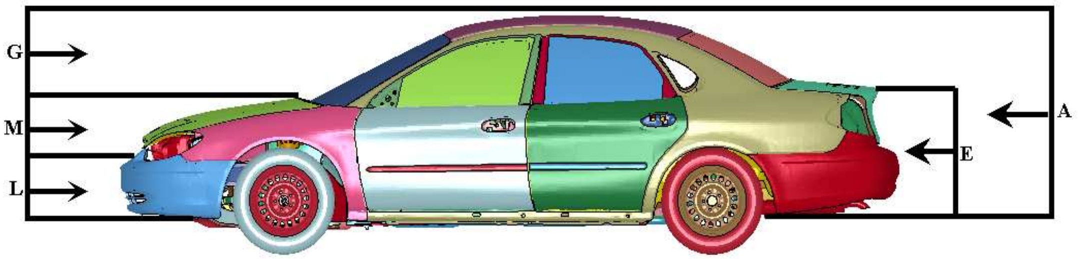

Figure 3. Specific vertical location of damage

BMJ Open

Associations between damage location and five main body regions injuries of MAIS 3-6 injured occupants

Journal:	*BMJ Open*
Manuscript ID:	bmjopen-2013-004371.R1
Article Type:	Research
Date Submitted by the Author:	28-Mar-2014
Complete List of Authors:	tang, youming; Xiamen University of Technology, School of Mechanical and Automotive Engineering; Hunan University, State Key Laboratory of Advanced Design and Manufacture of Vehicle Body<br>Cao, Libo; Hunan University, State Key Laboratory of Advanced Design and Manufacture of Vehicle Body<br>kan, steven; The George Washington University, National Crash Analysis Center
**Primary Subject Heading**:	Public health
Secondary Subject Heading:	Public health
Keywords:	PUBLIC HEALTH, Health & safety < HEALTH SERVICES ADMINISTRATION & MANAGEMENT, ACCIDENT & EMERGENCY MEDICINE

SCHOLARONE™
Manuscripts

Associations between damage location and five main body regions injuries of MAIS 3-6 injured occupants

Youming Tang,[1, 2, 3] Libo Cao,[2] Steven Kan[3]

[1] School of Mechanical and Automotive Engineering, Xiamen University of Technology, Xiamen, Fujian, China

[2]State Key Laboratory of Advanced Design and Manufacture for Vehicle Body, Hunan University, Changsha, Hunan, China

[3]National Crash Analysis Centre, The George Washington University, Ashburn, Virginia, USA

Correspondence to Youming Tang, School of Mechanical and Automotive Engineering, Xiamen University of Technology, Xiamen, Fujian, 361024, China; tangyouming@xmut.edu.cn or tangyouming.academic@gmail.com

ABSTRACT

Objectives: To estimate the overall maximum Abbreviated Injury Score (MAIS) 3-6 injured occupants of near-side struck vehicle which were associated with specific location of damage in front-to-side impact crashes.

Design and setting: MAIS 3-6 injured occupants information was extracted from the US-National Automotive Sampling System/ Crashworthiness Data System (NASS/CDS) in the year 2007, included head/face/neck, chest, pelvis, upper extremity and lower extremity. Struck vehicle collision damage was classified in a three-dimensional system according to J224 Collision Deformation Classification of SAE Surface Vehicle Standard.

Participants: Near-side occupants seated directly adjacent to the struck-side of the vehicle with MAIS 3-6 injured, in LTV-PC side impact crashes.

Outcome measures: Distribution of MAIS 3-6 injured occupants by body regions and specific location of damage (lateral direction, horizontal direction and vertical direction) were examined. Injury risk ratio was also assessed.

Results: The lateral crush zone contributed to MAIS 3-6 injured occupants (n=705) and 50th percentile injury risks when extended into zone 3. When the crush extended to zone 4, the injury risk ratio of MAIS 3-6 injured occupants was approached to 81%. The horizontal crush zone contributed to highest injury risk ratio of MAIS 3-6 occupants were zone "D" and zone "Y", and the injury risk ratio were 25.4% and 36.9%, respectively. In contrast, the lowest injury risk ratio was 5.67% caused by zone "B". The vertical crush zone contributed to highest injury risk ratio of MAIS 3-6 occupants was zone "E", the injury risk ratio was 58%. In contrast, the lowest injury risk ratio was 0.14% caused by zone "G+M".

Conclusions: The highest injury risk ratio of MAIS 3-6 injured occupants caused by crush intrusion between 40 centimeters and 60 centimeters in LTV-PC near-side impact collisions, and the damage region of struck vehicle were the zone "E" and "Y".

ARTICLE SUMMARY

Strengths and limitations of this study

- Gain a better understanding of stuck vehicle characteristics and crash conditions in the current and future side crash environment.
- The 50th percentile risk of MAIS 3-6 injuries to a front-to-side occupants between LTV and PC correspond to extent of damage in the zone 3, specific horizontal location of damage in the zone "Y", and specific vertical location of damage in the zone "E".
- To reduce the risk of deaths and fatal injuries of MAIS 3-6 injured occupants, considerate head, chest and pelvis injury prevention priority.
- The injury risk ratio of five main body regions of MAIS 0-2 injured occupants were excluded.

INTRODUCTION

Since 1980, there has been an increase in fatalities resulted from the collision of passenger cars (PC) and light truck vehicles (LTV), because LTV have became the majority of vehicles on the road, resulting in great occurrences of vehicle incompatibility between the body structures of LTV and PC.[1-4] LTV have the same truck frames include sport utility vehicles (SUV), pickup trucks, and vans weighted no more than 2041kg.[5]

For peer review only

The reinforcement of PC is most commonly a single beam at the mid-door or lower door cage, leaving the upper portion of door unprotected. When LTV collide with PC, the higher bumper frame contacts the door above the reinforcement, creating upper thoracic and head injuries.[6-7] Hence, vehicle mismatch between PC and LTV is associated with deaths and seriously injuries of occupants in automotive crashes.[8] In the present study, US national database was used to assess the near-side impact injury risks of five main body regions of maximum Abbreviated Injury Score (MAIS) 3-6 injured occupants between PC and LTV.

MATERIAL AND METHOD

Database of national automotive sampling system/ crashworthiness data system

The objective was to estimate the injury risks of MAIS 3-6 injured occupants by extent of damage for impacted vehicle in front-to-side impact crashes used real-world crash cases extracted from database of National Automotive Sampling System/ Crashworthiness Data System (NASS/CDS). The NASS/CDS is a detailed representative sample of motor vehicle crashes that occur within the United States. Each year roughly 5,000 detailed crash investigations are conducted by NASS investigators. Each case in the sample is assigned a weighting factor, which is equivalent to the inverse probability of such a vehicle incident occurring in a given year. Crashes occurred in the year 2007 were selected; the number of crash cases is 4,963 and after weighted is 2,454,014 collected by National Highway Traffic Safety Administration (NHTSA).

Selection criteria

In order to extract relevant data from the NASS/CDS datasets, the following selection criteria were established.

- Cases which only two vehicles were involved in the collision.
- Cases that resulted in a vehicle rollover were excluded.
- Crash configuration involved a front-to-side impact.
- Both of the vehicles involved could be classified as passenger car, and light truck vehicle including mini-van, sport utility vehicle, van, and light pickup truck.
- Striking vehicle was defined as an impact which resulted in the primary general area of damage being located at the front of the vehicle, also defined as having a direction of force between 330^0 and 30^0 (11'o clock to 1'o clock).
- Struck vehicle was defined as an impact which resulted in the primary general area of damage being located on either the right or the left side of the vehicle, also defined as having either a direction of force between 30^0 and 150^0 (1'o clock to 5'o clock right side impact), or between 210^0 and 330^0 (11'o clock to 7'o clock left side impact).
- Injury analysis was conducted base those injuries sustained by the near-side occupants seated directly adjacent to the struck-side of the vehicle, in a LTV-PC side impact crashes.

Collision deformation classification

According to the "J224 Collision Deformation Classification" of SAE surface vehicle standard, the extent of residual deformation induced by a vehicle was represented from horizontal, lateral and vertical direction.[9] For initially vehicle, the extent of lateral damage is classified using a nine-zone extent system relative to specific points on the vehicle structure. The extent of damage recorded, is dependent on the maximum cross-section to which the deformation extends to within a vehicle as a result of an impact. This measure also can be used to indicate the vehicle severity of an impact.

Vehicle horizontal areas to be used in locating the deformation for three independent extents and three composite extents, such as zone "P", "F", "B" and "Y", "Z", "D", respectively. "F" and "B" are side deformation areas forward and rearward of "P", respectively.

The classifications for the vertical location of deformations associated with impacts classified as "A", "E", "G", "M", or "L". The extents zone "A" and "E" are used for vehicle with both side deformation located rear-end, while "G", "M", and "L" located front-end. Zone "A" is used to classify impacts where the vehicle deformation resulted from an overhanging structure shaped like an inverted step in which the vertical

For peer review only

surfaces are at least 760 millimeters apart.[9]

RESULTS

PC-LTV crashes

The information presented in table 1 show that about 56% of road vehicle crashes resulted from vehicle-to-vehicle impact. These crashes also caused about 62% of injuries probability to overall occupants. As it can be seen, vehicle-to-vehicle crashes result in more seriously injuries probability per collision than single-vehicle crashes and multiple-vehicle crashes.

Table 2 shows the injury risks ratio of MAIS 3-6 injured occupants involved in two vehicle front-to-side crashes. The highest risk ratio of MAIS 3-6 injuries was 1 versus 2.09, which resulted from PC-to-PC side impact crashes. Comparatively the LTV-to-LTV side impact crashes caused the lowest injury risk ratio for MAIS 3-6 injured occupants.

Table 1 Investigation of road vehicle crash types in US in 2007

crash type	weighted-cases	weighted-occupants (not including pedestrians)
single-vehicle	832,385 （33.9%）	1,198,553 （19.9%）
vehicle-to-vehicle	1,361,888 （55.5%）	3,753,285 （62.2%）
multiple-vehicle	259,741 （10.6%）	1,082,825 （17.9%）
total	2,454,014 （100%）	6,034,663 （100%）

Table 2 MAIS 3-6 injured occupants involved in two passenger vehicle front-to-side crashes

crash configuration	injury risk ratio of MAIS 3-6 injured occupants (striking vehicle/struck vehicle)	injury risk ratio of MAIS 3-6 injured drivers (striking vehicle/struck vehicle)
PC-to-PC	1 (2,088): 2.09 (4,354)	1 (2,072): 2.05 (4,242)
PC-to-LTV	2.06 (667): 1 (324)	2.77 (653): 1 (236)
LTV-to-PC	1 (1,573): 1.84 (2,897)	1 (1,360): 2.13 (2,892)
LTV-to-LTV	1 (1,398): 1.49 (2,081)	1 (1,185): 1.32(1,565)
total	1 (5,726): 1.69 (9,656)	1 (5,270): 1.70 (8,935)

Specific lateral location of damage

Approximately 31% (95% C.I. 29.99%-32.07%) of MAIS 3-6 injuries were covered the extent zone 4 of damage. Zone 2 and zone 3 were comprised of only 20.4% (95% C.I. 19.39%-21.43%) and 27.5% (95% C.I. 26.44%-28.52%) of MAIS 3-6 injuries (table 3). As it can be seen, the crush zone attributed to occupants serious injured were zone 2, zone 3 and zone 4. 50th percentile of MAIS 3-6 injuries occurred when extent of damage crush into zone 3 of the struck vehicle.

The information in table 3 shows that the majority of AIS 3-6 injuries for head/face/neck (36.2%), chest (26.58%) and lower extremity (32.13%) were associated with an extent of damage in zone 4, while pelvis (45.51%), upper extremity (34.41%) were associated with an extent of damage in zone 3.The extent of damage in zones 3 and 4 accounted for about 56.87% of head/face/neck injuries, about 49.65% of chest injuries, about 68.2% of pelvis injuries, about 65.6% of upper extremity injuries and about 62.39% of lower extremity injuries, respectively.

Assuming a passenger car's width was 1.8 meters in LTV-to-PC near-side impact crashes, the each extent of damage was 20 centimeters. Zone 1 demonstrated crush intrusion in lateral location of damage was between 0 and 20 centimeters, zone 2 demonstrated crush intrusion was between 20 centimeters and 40 centimeters... zone 9 demonstrated crush intrusion was between 160 centimeters and 180 centimeters and included broken crush, according to J224 Collision Deformation Classification of SAE Surface Vehicle Standard. However, the distance commonly between occupants and interior panel is less than 20 centimeters. Therefore, crush intrusion of vehicle side components is almost up to 60 centimeters will cause the occupants to lose capacitating or even fatal injury. In current study, the risk ratio of MAIS 3-6 injuries was 80.5% high resulted from the extent of damage extended into zone 4.

For peer review only

Table 3 Distribution of MAIS 3-6 injured occupants by body regions and lateral location of damage in US in 2007 (N=564, Missing data=141)

lateral location of damage<br>95% Confidential Interval		1	2	3	4	5	6	7	8	9
occupants number	Percentage	1.6%	20.39%	27.48%	31.03%	14.54%	4.96%	0	0	0
		(0.56-2.64)	(19.39-21.43)	(26.44-28.52)	(29.99-32.07)	(13.5-15.58)	(3.92-6)	(0-1.04)	(0-1.04)	(0-1.04)
	Cumulative percentage	1.6%	21.99%	49.47%	80.5%	95.04%	100%	100%	100%	100%
Five Main body regions	head/face/neck	3.47%	23.56%	20.67%	36.2%	14.94%	1.17%	0	0	0
		1.07 (0.84-1.3)	7.27 (7.04-7.5)	6.38 (6.15-6.61)	11.17(10.94-11.4)	4.61(4.38-4.84)	0.36(0.13-0.59)	0(0-0.23)	0(0-0.23)	0(0-0.23)
	chest	0	18.89%	23.07%	26.58%	23.78%	7.69%	0	0	0
		0 (0-0.23)	4.79(4.56-5.02)	5.85 (5.62-6.08)	6.74 (6.51-6.97)	6.03(5.8-6.26)	1.95(1.72-2.18)	0(0-0.23)	0(0-0.23)	0(0-0.23)
	pelvis	0	25%	45.51%	22.69%	4.49%	2.31%	0	0	0
		0 (0-0.23)	1.95 (1.72-2.18)	3.55 (3.32-3.78)	1.77 (1.54-2)	0.35(0.12-0.58)	0.18(0-0.41)	0(0-0.23)	0(0-0.23)	0(0-0.23)
	upper extremity	2.12%	18.26%	34.41%	31.19%	9.71%	4.31%	0	0	0
		0.35(0.12-0.58)	3.01(2.78-3.24)	5.67(5.44-5.9)	5.14 (4.91-5.37)	1.6(1.37-1.83)	0.71(0.48-0.94)	0(0-0.23)	0(0-0.23)	0(0-0.23)
	lower extremity	0.93%	17.43%	30.26%	32.13%	10.09%	9.16%	0	0	0
		0.18(0-0.41)	3.37(3.14-3.6)	5.85(5.62-6.08)	6.21 (5.98-6.44)	1.95(1.72-2.18)	1.77(1.54-2)	0(0-0.23)	0(0-0.23)	0(0-0.23)

Specific horizontal location of damage

The two highest injury ratios of MAIS 3-6 injuries were 25.39% (95% C.I. 24.46%-26.32%) and 36.88% (95% C.I. 35.95%-37.81%) respectively covered the damage location "D" and "Y" (table 4); comprised the lowest injury ratio of MAIS 3-6 injuries was only 5.67% (95% C.I. 4.74%-6.6 %) covered the damage location "B".

Over half of all body AIS 3-6 injuries were contributed to the damage location "D" and "Y". The highest risk ratio of MAIS 3-6 injuries for head/face/neck, chest, pelvis, upper extremity and lower extremity was respectively about 35.95%, 36.99%, 25.98%, 40% and 47.88% associated with the damage zone "Y" and zone "D". In contrast, the lowest risk ratio of MAIS 3-6 injuries for head/face/neck, chest, pelvis, upper extremity and lower extremity was only about 6.48%, 4.07%, 3.66%, 2.64% and 5.3% respectively associated with the damage zone "B".

Table 4 Distribution of MAIS 3-6 injured occupants by body regions and horizontal location of damage in US in 2007 (N=705)

horizontal location of damage<br>95% Confidential Interval		unknown	B	D	F	P	Y	Z
occupants number	Percentage	0.14%(0-1.07)	5.67%	25.39%	10.35%	8.94%	36.88%	12.62%
			(4.74-6.6)	(24.46-26.32)	(9.42-11.28)	(8.01-9.87)	(35.95-37.81)	(11.69-13.55)
five main body regions	head/face/neck	0	7.79%	22.97%	12.98%	6.48%	35.95%	13.84%
		0 (0-0.22)	2.55(2.33-2.77)	7.52(7.3-7.74)	4.25(4.03-4.47)	2.12(1.9-2.34)	11.77(11.55-11.99)	4.53(4.31-4.75)
	chest	0.57%	4.07%	36.99%	6.92%	9.82%	30.63%	11%
		0.14(0-0.36)	1(0.78-1.22)	9.08(8.86-9.3)	1.7(1.48-1.92)	2.41(2.19-2.63)	7.52(7.3-7.74)	2.7(2.48-2.92)
	pelvis	0	9.27%	16.71%	20.37%	3.66%	25.98%	24.02%
		0 (0-0.22)	0.71(0.49-0.93)	1.28(1.06-1.5)	1.56(1.34-1.78)	0.28(0.06-0.5)	1.99(1.77-2.21)	1.84(1.62-2.06)
	upper extremity	0	2.64%	20%	11.29%	10.43%	40%	15.64%
		0 (0-0.22)	0.43(0.21-0.65)	3.26(3.04-3.48)	1.84(1.62-2.06)	1.7(1.48-1.92)	6.52(6.3-6.74)	2.55(2.33-2.77)

For peer review only

lower extremity	0.48%	5.3%	22.82%	5.3%	12.91%	47.88%	5.3%
	0.09(0-0.31)	0.99(0.77-1.21)	4.26(4.04-4.48)	0.99(0.77-1.21)	2.41(2.19-2.63)	8.94(8.72-9.16)	0.99(0.77-1.21)

Specific vertical location of damage

Approximately 57.6% (95% C.I. 55.57%-59.61%) of MAIS 3-6 injuries were resulted from the damage location "E" (table 5); zone "G+M" was comprised of only 0.14% (95% C.I. 0-2.16%) of MAIS 3-6 injuries.

As it can be seen, the majority of all MAIS 3-6 injuries were contribute to the damage location "E", the risk ratio of MAIS 3-6 injuries for head/face/neck, chest, pelvis, upper extremity and lower extremity was about 55.4%, 50.26%, 70.37%, 71.26% and 60.3%, respectively.

Table 5 Distribution of MAIS 3-6 injured occupants by body regions and vertical location of damage in US in 2007 (N=705)

vertical location of damage / 95% Confidential Interval		unknown	A	E	G+M	L
occupants number	Percentage	4.82%(2.8-6.84)	37.45%(35.43-39.47)	57.59%(55.57-59.61)	0.14%(0-2.16)	0(0-2.08)
five main body regions	head/face/neck	6.5%	38.1%	55.4%	0	0
		2.13(1.72-2.54)	12.48(12.07-12.89)	18.15(17.74-18.56)	0 (0-0.41)	0 (0-0.43)
	chest	4.07%	50.26%	45.09%	0.57%	0
		1(0.57-1.43)	12.34(11.91-12.77)	11.07(10.64-11.5)	0.14(0-0.57)	0 (0-0.43)
	pelvis	7.44%	22.19%	70.37%	0	0
		0.57(0.14-1)	1.7(1.27-2.13)	5.39(4.96-5.82)	0 (0-0.43)	0 (0-0.43)
	upper extremity	2.63%	26.1%	71.26%	0	0
		0.43(0-0.86)	4.26(3.83-4.69)	11.63(11.2-12.06)	0 (0-0.43)	0 (0-0.43)
	lower extremity	3.82%	35.88%	60.3%	0	0
		0.71(0.28-1.14)	6.67(6.24-7.1)	11.21(10.78-11.64)	0 (0-0.43)	0 (0-0.43)

12-14

DISCUSSION

We evaluated the risks of MAIS 3-6 injuries of head/face/neck, chest, pelvis, upper extremity and lower extremity from two passenger vehicle front-to-side impact crashes. Our analysis was based upon an examination of approximately 1,362,000 weighted cases of PC and LTV which were extracted from the US-NASS/CDS 2007 crash investigations database.

Previous studies have indicated occupants of PC were more likely than occupants of LTV to be seriously injured. About 2.9% of PC occupants and 1.9% of LTV occupants in side-impacted vehicles were seriously injured.[10] Crash aggressivity index (CAI) of LTV, compared to the reference case PC (0.00), the increased from vans (0.32) until medium truck (0.48), and then for heavy truck (0.40) and truck tractor (0.21) it decreased.[11] PC-LTV incompatibility in vehicle mass, vehicle geometry and structure also have been widely discussed.

In our study, the injuries risk ratios in front-to-side impact crashes was that overall occupants of struck vehicle have a sixty-nine percent higher MAIS 3-6 injury risks than overall occupants of striking vehicle. The reason is that the striking vehicle occupants are, to a greater extent, protected by the presence of a larger structural crush zone, i.e. including the energy-absorbing bumper and front-end structure, the vehicle's engine, the front suspension and wheels, the engine-mounting frame and integral firewall, supplemental safety systems. However, the struck vehicle crush zone is comprised of only the side doors and the relatively light framework of the occupant cell, plus interior foams which offer some limited protection, or some vehicle types are now also equipped with supplemental side airbags or curtains which have increased the level of near-side impact protection available to the occupants, but the level of protection does not reach the level which currently exists

For peer review only - http://bmjopen.bmj.com/site/about/guidelines.xhtml

for frontal crashes.

The results from lateral damage location suggest that strategies and methodologies of occupant protection and injuries reduction are developed from the extent of damage in zones 1, 2 and 3. Two methods are often used to improve vehicle safety in near-side impact crashes. Method number one is enhancing the structural rigidity of interior panel of side doors to reduce magnitude of door panel crush, such as optimized design of impact bar, foam systems and door interior panels. Method number two is adding the airbags between the occupant and interior panel, i.e. supplemental head curtains and chest airbags which have increased the level of protection available to occupants. Tencer et al. (2005) showed that magnitude of door crush in near-side impact from category 1 (3-8 centimeters of crush) to category 2 (9-15 centimeters of crush) was associated with an average of 0.4 increase in thoracic AIS, a 0.3 increase in abdominal AIS, and a 0.2 increase in pelvis (p<0.0001).[6] In our data, there was not consideration of crush intrusion on MAIS below 3. We analyzed the magnitude of side crush intrusion in near-side impact from zone 2 (about 20-40 centimeters of crush) to zone 3 (about 40-60 centimeters of crush) was associated with an average of 0.22 increase in chest MAIS 3-6, a 0.82 increase in pelvis MAIS 3-6, and a 0.88 increase in upper extremity MAIS 3-6 (p<0.0001).

The results from horizontal damage location suggest that the primary crushed location of impacted vehicle is forward of c-pillar in real world near-side impact crashes, and it is less possibility to impact the rearward of c-pillar for target vehicle. Therefore, for design researches it is useful to maintain the side rigidity of vehicle specially accounted for structure rigidity of side doors, A-pillar, B-pillar and rocker rail.

The results from vertical damage location suggest that the primary crushed location of impacted vehicle is located in upward of rocker rail and downward of windowsill in real world near-side impact crashes, and it is less possibility to impact the upward of windowsill for target vehicle. Considering improving the level of side protection particularly should enhance the rigidity of side doors and rocker rail from perspective of vertical anti-crushed location of vehicle, or consider vehicle front end design, especially for LTV, in motor vehicle safety standards.[15]

In conclusion, over half of crash types in real world are attributed to vehicle-to-vehicle crashes. Rigidity of side doors, A-pillar, B-pillar and rocker rail are priorities for improving the safety level of near-side impact. Because the specific location of damage accounted for MAIS 3-6 injured occupants and 50% injury risk is extent zone 3, zone “Y” and “E” from lateral, horizontal and vertical direction of struck vehicle, respectively. To reduce the risk of deaths and serious injury, protection of head, chest and pelvis should be priorities for injury countermeasure development, because these three body regions accounted for over half of AIS 3-6 injuries suffered from vertical crush zone “E” in PC-LTV near-side impact crashes.

Acknowledgements We would like to thank Dr. Prodeep Mohan (senior scientist of National Crash Analysis Center, George Washington University), Ms. Christina (master graduate student of National Crash Analysis Center, George Washington University), and Mr. Leyu Wang (doctoral graduate student of National Crash Analysis Center, George Washington University) for their very valuable suggestions and software processing to this work.

Contributors YT was the primary investigator, he initiated and designed the study and completed the data processing and manuscript writing. LC and SK participated in developing the study concept and data interpretation. All authors reviewed and approved the final manuscript.

Funding This project was supported by National Crash Analysis Center (NCAC), the George Washington University (GWU) and sponsored by National Natural Science Foundation of China, grant no.51305374 and no.51355001. Open Foundation of State Key Laboratory of Advanced Design and Manufacture for Vehicle Body of Hunan University, grant no. 31215004. Fujian Natural Science Foundation of China, grant no. 2012J05103.

Competing Interests: None

Data Sharing Statement: Extra data is available by emailing tangyouming@xmut.edu.cn

Competing interests None.

For peer review only

REFERENCES

1. **Acierno S**, Kaufman R, Rivara FP, at al. Vehicle mismatch: injury patterns and severity. *Accid Anal and Prev* 2004; **36**: 761-772.
2. **Bradsher K**. The triumph of SUVs. *High and mighty*. 1st Ed. New York: Public Affairs, 2001:382-412
3. **Samaha RR**, Elliott DS. NHTSA side impact research: motivation for upgraded test procedures. Nagoya, Japan, May 2003. *18th Conference on the Enhanced Safety of Vehicles (ESV)* 2003; paper No.492:1-17
4. **Mandell SP**, Mack CD and Bulger EM. Moter vehicle mismatch: a national perspective. *Inj Prev* 2010; **16**: 309-314.
5. **Crashworthiness Data System** (CDS) 2007 coding and editing manual. Washington D.C: Transportation National Highway Traffic Safety Administration, 2007.
6. **Tencer AF**, Kaufman R, Mack C, et al. Factors affecting pelvic and thoracic forces in near side impact crashes: a study of US-NCAP, NASS and CIREN data. *Accid Anal and Prev* 2005; **37**: 287-293.
7. **Insurance Institute for Highway Safety** (IIHS). Putting the crash compatibility issue in perspective. *Insurance Institute for Highway Safety Status Rep*ort 1999; 34:1–11.
8. **Eric MO,** Thomas DK and Barbara M. Will the light truck bumper height-matching standard reduce deaths in cars?. *Traf Inj Prev* 2013; **51**: 292-300.
9. **Society of Automotive Engineering**. *Collision deformation classification of surface vehicle standard-1980*. Warrendale: SAE, 1980.
10. **Charles MF**, Elisa RB and Eric LM. Two-vehicle side impact crashes: the relationship of vehicle and crash characteristics to injury severity. *Accid Anal and Prev* 1997;**29**: 399-406.
11. **Helai H**, Chowdhury S and Mohamed AA. Indexing crash worthiness and crash aggressivity by vehicle type. *Accid Anal and Prev* 2011; **43**: 1364-1370
12. **Toy EL** and Hammitt JK. Safety impacts of suvs, minivans, and pickup trucks in two-vehicle crashes. *Risk Anal* 2003; **23**: 641-650.
13. **Kahane CJ**. Vehicle weight, fatality risk and crash compatibility of model year 1991-99 passenger cars and light trucks. *NHTSA Technical Report* No. DOT HS 809 662, 2003.
14. **Fredette M**, Mambu LS, Chouinard A, at al. Safety impacts due to the incompatibility of suvs, minivans, and pickup trucks in two-vehicle collisions. *Accid Anal and Prev* 2008; 40:1987-1995
15. **Roudsari BS**, Mork CN, Kaufman R, et al. Pedestrian crashes: higher injury severity and mortality rate for light truck vehicles compared with passenger vehicles. *Inj Prev* 2004; **10**:154-158

For peer review only

Associations between damage location and five main body regions injuries of MAIS 3-6 injured occupants

Youming Tang,[1, 2, 3] Libo Cao,[2] Steven Kan[3]

[1] School of Mechanical and Automotive Engineering, Xiamen University of Technology, Xiamen, Fujian, China

[2]State Key Laboratory of Advanced Design and Manufacture for Vehicle Body, Hunan University, Changsha, Hunan, China

[3]National Crash Analysis Centre, The George Washington University, Ashburn, Virginia, USA

Correspondence to Youming Tang, School of Mechanical and Automotive Engineering, Xiamen University of Technology, Xiamen, Fujian, 361024, China; tangyouming@xmut.edu.cn or tangyouming.academic@gmail.com

ABSTRACT

Objectives: To estimate the overall maximum Abbreviated Injury Score (MAIS) 3-6 injured occupants of near-side struck vehicle which were associated with specific location of damage in front-to-side impact crashes.

Design and setting: MAIS 3-6 injured occupants information was extracted from the US-National Automotive Sampling System/ Crashworthiness Data System (NASS/CDS) in the year 2007, included head/face/neck, chest, pelvis, upper extremity and lower extremity. Struck vehicle collision damage was classified in a three-dimensional system according to J224 Collision Deformation Classification of SAE Surface Vehicle Standard.

Participants: Near-side occupants seated directly adjacent to the struck-side of the vehicle with MAIS 3-6 injured, in LTV-PC side impact crashes.

Outcome measures: Distribution of MAIS 3-6 injured occupants by body regions and specific location of damage (lateral direction, horizontal direction and vertical direction) were examined. Injury risk ratio was also assessed.

Results: The lateral crush zone contributed to MAIS 3-6 injured occupants (n=705) and 50th percentile injury risks when extended into zone 3. When the crush extended to zone 4, the injury risk ratio of MAIS 3-6 injured occupants was approached to 81%. The horizontal crush zone contributed to highest injury risk ratio of MAIS 3-6 occupants were zone "D" and zone "Y", and the injury risk ratio were 25.4% and 36.9%, respectively. In contrast, the lowest injury risk ratio was 5.67% caused by zone "B". The vertical crush zone contributed to highest injury risk ratio of MAIS 3-6 occupants was zone "E", the injury risk ratio was 58%. In contrast, the lowest injury risk ratio was 0.14% caused by zone "G+M".

Conclusions: The highest injury risk ratio of MAIS 3-6 injured occupants caused by crush intrusion between 40 centimeters and 60 centimeters in LTV-PC near-side impact collisions, and the damage region of struck vehicle were the zone "E" and "Y".

ARTICLE SUMMARY

Strengths and limitations of this study

- Gain a better understanding of stuck vehicle characteristics and crash conditions in the current and future side crash environment.
- The 50th percentile risk of MAIS 3-6 injuries to a front-to-side occupants between LTV and PC correspond to extent of damage in the zone 3, specific horizontal location of damage in the zone "Y", and specific vertical location of damage in the zone "E".
- To reduce the risk of deaths and fatal injuries of MAIS 3-6 injured occupants, considerate head, chest and pelvis injury prevention priority.
- The injury risk ratio of five main body regions of MAIS 0-2 injured occupants were excluded.

INTRODUCTION

Since 1980, there has been an increase in fatalities resulted from the collision of passenger cars (PC) and light truck vehicles (LTV), because LTV have became the majority of vehicles on the road, resulting in great occurrences of vehicle incompatibility between the body structures of LTV and PC.[1-4] LTV have the same truck frames include sport utility vehicles (SUV), pickup trucks, and vans weighted no more than 2041kg.[5]

For peer review only

The reinforcement of PC is most commonly a single beam at the mid-door or lower door cage, leaving the upper portion of door unprotected. When LTV collide with PC, the higher bumper frame contacts the door above the reinforcement, creating upper thoracic and head injuries.[6-7] Hence, vehicle mismatch between PC and LTV is associated with deaths and seriously injuries of occupants in automotive crashes.[8] In the present study, US national database was used to assess the near-side impact injury risks of five main body regions of maximum Abbreviated Injury Score (MAIS) 3-6 injured occupants between PC and LTV.

MATERIAL AND METHOD

Database of national automotive sampling system/ crashworthiness data system

The objective was to estimate the injury risks of MAIS 3-6 injured occupants by extent of damage for impacted vehicle in front-to-side impact crashes used real-world crash cases extracted from database of National Automotive Sampling System/ Crashworthiness Data System (NASS/CDS). The NASS/CDS is a detailed representative sample of motor vehicle crashes that occur within the United States. Each year roughly 5,000 detailed crash investigations are conducted by NASS investigators. Each case in the sample is assigned a weighting factor, which is equivalent to the inverse probability of such a vehicle incident occurring in a given year. Crashes occurred in the year 2007 were selected; the number of crash cases is 4,963 and after weighted is 2,454,014 collected by National Highway Traffic Safety Administration (NHTSA).

Selection criteria

In order to extract relevant data from the NASS/CDS datasets, the following selection criteria were established.

- Cases which only two vehicles were involved in the collision.
- Cases that resulted in a vehicle rollover were excluded.
- Crash configuration involved a front-to-side impact.
- Both of the vehicles involved could be classified as passenger car, and light truck vehicle including mini-van, sport utility vehicle, van, and light pickup truck.
- Striking vehicle was defined as an impact which resulted in the primary general area of damage being located at the front of the vehicle, also defined as having a direction of force between 330^0 and 30^0 (11'o clock to 1'o clock).
- Struck vehicle was defined as an impact which resulted in the primary general area of damage being located on either the right or the left side of the vehicle, also defined as having either a direction of force between 30^0 and 150^0 (1'o clock to 5'o clock right side impact), or between 210^0 and 330^0 (11'o clock to 7'o clock left side impact).
- Injury analysis was conducted base those injuries sustained by the near-side occupants seated directly adjacent to the struck-side of the vehicle, in a LTV-PC side impact crashes.

Collision deformation classification

According to the "J224 Collision Deformation Classification" of SAE surface vehicle standard, the extent of residual deformation induced by a vehicle was represented from horizontal, lateral and vertical direction.[9] For initially vehicle, the extent of lateral damage is classified using a nine-zone extent system relative to specific points on the vehicle structure. The extent of damage recorded, is dependent on the maximum cross-section to which the deformation extends to within a vehicle as a result of an impact. This measure also can be used to indicate the vehicle severity of an impact.

Vehicle horizontal areas to be used in locating the deformation for three independent extents and three composite extents, such as zone "P", "F", "B" and "Y", "Z", "D", respectively. "F" and "B" are side deformation areas forward and rearward of "P", respectively.

The classifications for the vertical location of deformations associated with impacts classified as "A", "E", "G", "M", or "L". The extents zone "A" and "E" are used for vehicle with both side deformation located rear-end, while "G", "M", and "L" located front-end. Zone "A" is used to classify impacts where the vehicle deformation resulted from an overhanging structure shaped like an inverted step in which the vertical

For peer review only

surfaces are at least 760 millimeters apart.[9]

RESULTS

PC-LTV crashes

The information presented in table 1 show that about 56% of road vehicle crashes resulted from vehicle-to-vehicle impact. These crashes also caused about 62% of injuries probability to overall occupants. As it can be seen, vehicle-to-vehicle crashes result in more seriously injuries probability per collision than single-vehicle crashes and multiple-vehicle crashes.

Table 2 shows the injury risks ratio of MAIS 3-6 injured occupants involved in two vehicle front-to-side crashes. The highest risk ratio of MAIS 3-6 injuries was 1 versus 2.09, which resulted from PC-to-PC side impact crashes. Comparatively the LTV-to-LTV side impact crashes caused the lowest injury risk ratio for MAIS 3-6 injured occupants.

Table 1 Investigation of road vehicle crash types in US in 2007

crash type	weighted-cases	weighted-occupants (not including pedestrians)
single-vehicle	832,385 （33.9%）	1,198,553 （19.9%）
vehicle-to-vehicle	1,361,888 （55.5%）	3,753,285 （62.2%）
multiple-vehicle	259,741 （10.6%）	1,082,825 （17.9%）
total	2,454,014 （100%）	6,034,663 （100%）

Table 2 MAIS 3-6 injured occupants involved in two passenger vehicle front-to-side crashes

crash configuration	injury risk ratio of MAIS 3-6 injured occupants (striking vehicle/struck vehicle)<br>injury risk ratio of MAIS 3-6 injured drivers (striking vehicle/struck vehicle)
PC-to-PC	1 (2,088): 2.09 (4,354)<br>1 (2,072): 2.05 (4,242)
PC-to-LTV	2.06 (667): 1 (324)<br>2.77 (653): 1 (236)
LTV-to-PC	1 (1,573): 1.84 (2,897)<br>1 (1,360): 2.13 (2,892)
LTV-to-LTV	1 (1,398): 1.49 (2,081)<br>1 (1,185): 1.32(1,565)
total	1 (5,726): 1.69 (9,656)<br>1 (5,270): 1.70 (8,935)

Specific lateral location of damage

Approximately 31% (95% C.I. 29.99%-32.07%) of MAIS 3-6 injuries were covered the extent zone 4 of damage. Zone 2 and zone 3 were comprised of only 20.4% (95% C.I. 19.39%-21.43%) and 27.5% (95% C.I. 26.44%-28.52%) of MAIS 3-6 injuries (table 3). As it can be seen, the crush zone attributed to occupants serious injured were zone 2, zone 3 and zone 4. 50th percentile of MAIS 3-6 injuries occurred when extent of damage crush into zone 3 of the struck vehicle.

The information in table 3 shows that the majority of AIS 3-6 injuries for head/face/neck (36.2%), chest (26.58%) and lower extremity (32.13%) were associated with an extent of damage in zone 4, while pelvis (45.51%), upper extremity (34.41%) were associated with an extent of damage in zone 3.The extent of damage in zones 3 and 4 accounted for about 56.87% of head/face/neck injuries, about 49.65% of chest injuries, about 68.2% of pelvis injuries, about 65.6% of upper extremity injuries and about 62.39% of lower extremity injuries, respectively.

Assuming a passenger car's width was 1.8 meters in LTV-to-PC near-side impact crashes, the each extent of damage was 20 centimeters. Zone 1 demonstrated crush intrusion in lateral location of damage was between 0 and 20 centimeters, zone 2 demonstrated crush intrusion was between 20 centimeters and 40 centimeters... zone 9 demonstrated crush intrusion was between 160 centimeters and 180 centimeters and included broken crush, according to J224 Collision Deformation Classification of SAE Surface Vehicle Standard. However, the distance commonly between occupants and interior panel is less than 20 centimeters. Therefore, crush intrusion of vehicle side components is almost up to 60 centimeters will cause the occupants to lose capacitating or even fatal injury. In current study, the risk ratio of MAIS 3-6 injuries was 80.5% high resulted from the extent of damage extended into zone 4.

Table 3 Distribution of MAIS 3-6 injured occupants by body regions and lateral location of damage in US in 2007 (N=564, Missing data=141)

For peer review only

lateral location of damage<br>95% Confidential Interval		1	2	3	4	5	6	7	8	9
occupants number	Percentage	1.6%<br>(0.56-2.64)	20.39%<br>(19.39-21.43)	27.48%<br>(26.44-28.52)	31.03%<br>(29.99-32.07)	14.54%<br>(13.5-15.58)	4.96%<br>(3.92-6)	0<br>(0-1.04)	0<br>(0-1.04)	0<br>(0-1.04)
	Cumulative percentage	1.6%	21.99%	49.47%	80.5%	95.04%	100%	100%	100%	100%
Five Main body regions	head/face/neck	3.47%<br>1.07 (0.84-1.3)	23.56%<br>7.27 (7.04-7.5)	20.67%<br>6.38 (6.15-6.61)	36.2%<br>11.17(10.94-11.4)	14.94%<br>4.61(4.38-4.84)	1.17%<br>0.36(0.13-0.59)	0<br>0(0-0.23)	0<br>0(0-0.23)	0<br>0(0-0.23)
	chest	0<br>0 (0-0.23)	18.89%<br>4.79(4.56-5.02)	23.07%<br>5.85 (5.62-6.08)	26.58%<br>6.74 (6.51-6.97)	23.78%<br>6.03(5.8-6.26)	7.69%<br>1.95(1.72-2.18)	0<br>0(0-0.23)	0<br>0(0-0.23)	0<br>0(0-0.23)
	pelvis	0<br>0 (0-0.23)	25%<br>1.95 (1.72-2.18)	45.51%<br>3.55 (3.32-3.78)	22.69%<br>1.77 (1.54-2)	4.49%<br>0.35(0.12-0.58)	2.31%<br>0.18(0-0.41)	0<br>0(0-0.23)	0<br>0(0-0.23)	0<br>0(0-0.23)
	upper extremity	2.12%<br>0.35(0.12-0.58)	18.26%<br>3.01(2.78-3.24)	34.41%<br>5.67(5.44-5.9)	31.19%<br>5.14 (4.91-5.37)	9.71%<br>1.6(1.37-1.83)	4.31%<br>0.71(0.48-0.94)	0<br>0(0-0.23)	0<br>0(0-0.23)	0<br>0(0-0.23)
	lower extremity	0.93%<br>0.18(0-0.41)	17.43%<br>3.37(3.14-3.6)	30.26%<br>5.85(5.62-6.08)	32.13%<br>6.21 (5.98-6.44)	10.09%<br>1.95(1.72-2.18)	9.16%<br>1.77(1.54-2)	0<br>0(0-0.23)	0<br>0(0-0.23)	0<br>0(0-0.23)

Specific horizontal location of damage

The two highest injury ratios of MAIS 3-6 injuries were 25.39% (95% C.I. 24.46%-26.32%) and 36.88% (95% C.I. 35.95%-37.81%) respectively covered the damage location "D" and "Y" (table 4); comprised the lowest injury ratio of MAIS 3-6 injuries was only 5.67% (95% C.I. 4.74%-6.6 %) covered the damage location "B".

Over half of all body AIS 3-6 injuries were contributed to the damage location "D" and "Y". The highest risk ratio of MAIS 3-6 injuries for head/face/neck, chest, pelvis, upper extremity and lower extremity was respectively about 35.95%, 36.99%, 25.98%, 40% and 47.88% associated with the damage zone "Y" and zone "D". In contrast, the lowest risk ratio of MAIS 3-6 injuries for head/face/neck, chest, pelvis, upper extremity and lower extremity was only about 6.48%, 4.07%, 3.66%, 2.64% and 5.3% respectively associated with the damage zone "B".

Table 4 Distribution of MAIS 3-6 injured occupants by body regions and horizontal location of damage in US in 2007 (N=705)

horizontal location of damage<br>95% Confidential Interval		unknown	B	D	F	P	Y	Z
occupants number	Percentage	0.14%(0-1.07)	5.67%<br>(4.74-6.6)	25.39%<br>(24.46-26.32)	10.35%<br>(9.42-11.28)	8.94%<br>(8.01-9.87)	36.88%<br>(35.95-37.81)	12.62%<br>(11.69-13.55)
five main body regions	head/face/neck	0<br>0 (0-0.22)	7.79%<br>2.55(2.33-2.77)	22.97%<br>7.52(7.3-7.74)	12.98%<br>4.25(4.03-4.47)	6.48%<br>2.12(1.9-2.34)	35.95%<br>11.77(11.55-11.99)	13.84%<br>4.53(4.31-4.75)
	chest	0.57%<br>0.14(0-0.36)	4.07%<br>1(0.78-1.22)	36.99%<br>9.08(8.86-9.3)	6.92%<br>1.7(1.48-1.92)	9.82%<br>2.41(2.19-2.63)	30.63%<br>7.52(7.3-7.74)	11%<br>2.7(2.48-2.92)
	pelvis	0<br>0 (0-0.22)	9.27%<br>0.71(0.49-0.93)	16.71%<br>1.28(1.06-1.5)	20.37%<br>1.56(1.34-1.78)	3.66%<br>0.28(0.06-0.5)	25.98%<br>1.99(1.77-2.21)	24.02%<br>1.84(1.62-2.06)
	upper extremity	0<br>0 (0-0.22)	2.64%<br>0.43(0.21-0.65)	20%<br>3.26(3.04-3.48)	11.29%<br>1.84(1.62-2.06)	10.43%<br>1.7(1.48-1.92)	40%<br>6.52(6.3-6.74)	15.64%<br>2.55(2.33-2.77)
	lower extremity	0.48%<br>0.09(0-0.31)	5.3%<br>0.99(0.77-1.21)	22.82%<br>4.26(4.04-4.48)	5.3%<br>0.99(0.77-1.21)	12.91%<br>2.41(2.19-2.63)	47.88%<br>8.94(8.72-9.16)	5.3%<br>0.99(0.77-1.21)

For peer review only

Specific vertical location of damage

Approximately 57.6% (95% C.I. 55.57%-59.61%) of MAIS 3-6 injuries were resulted from the damage location "E" (table 5); zone "G+M" was comprised of only 0.14% (95% C.I. 0-2.16%) of MAIS 3-6 injuries.

As it can be seen, the majority of all MAIS 3-6 injuries were contribute to the damage location "E", the risk ratio of MAIS 3-6 injuries for head/face/neck, chest, pelvis, upper extremity and lower extremity was about 55.4%, 50.26%, 70.37%, 71.26% and 60.3%, respectively.

Table 5 Distribution of MAIS 3-6 injured occupants by body regions and vertical location of damage in US in 2007 (N=705)

vertical location of damage<br>95% Confidential Interval		unknown	A	E	G+M	L
occupants number	Percentage	4.82%(2.8-6.84)	37.45%(35.43-39.47)	57.59%(55.57-59.61)	0.14%(0-2.16)	0(0-2.08)
five main body regions	head/face/neck	6.5%	38.1%	55.4%	0	0
		2.13(1.72-2.54)	12.48(12.07-12.89)	18.15(17.74-18.56)	0 (0-0.41)	0 (0-0.43)
	chest	4.07%	50.26%	45.09%	0.57%	0
		1(0.57-1.43)	12.34(11.91-12.77)	11.07(10.64-11.5)	0.14(0-0.57)	0 (0-0.43)
	pelvis	7.44%	22.19%	70.37%	0	0
		0.57(0.14-1)	1.7(1.27-2.13)	5.39(4.96-5.82)	0 (0-0.43)	0 (0-0.43)
	upper extremity	2.63%	26.1%	71.26%	0	0
		0.43(0-0.86)	4.26(3.83-4.69)	11.63(11.2-12.06)	0 (0-0.43)	0 (0-0.43)
	lower extremity	3.82%	35.88%	60.3%	0	0
		0.71(0.28-1.14)	6.67(6.24-7.1)	11.21(10.78-11.64)	0 (0-0.43)	0 (0-0.43)

DISCUSSION

We evaluated the risks of MAIS 3-6 injuries of head/face/neck, chest, pelvis, upper extremity and lower extremity from two passenger vehicle front-to-side impact crashes. Our analysis was based upon an examination of approximately 1,362,000 weighted cases of PC and LTV which were extracted from the US-NASS/CDS 2007 crash investigations database.

Previous studies have indicated occupants of PC were more likely than occupants of LTV to be seriously injured. About 2.9% of PC occupants and 1.9% of LTV occupants in side-impacted vehicles were seriously injured.[10] Crash aggressivity index (CAI) of LTV, compared to the reference case PC (0.00), the increased from vans (0.32) until medium truck (0.48), and then for heavy truck (0.40) and truck tractor (0.21) it decreased.[11] PC-LTV incompatibility in vehicle mass, vehicle geometry and structure also have been widely discussed.[12-14]

In our study, the injuries risk ratios in front-to-side impact crashes was that overall occupants of struck vehicle have a sixty-nine percent higher MAIS 3-6 injury risks than overall occupants of striking vehicle. The reason is that the striking vehicle occupants are, to a greater extent, protected by the presence of a larger structural crush zone, i.e. including the energy-absorbing bumper and front-end structure, the vehicle's engine, the front suspension and wheels, the engine-mounting frame and integral firewall, supplemental safety systems. However, the struck vehicle crush zone is comprised of only the side doors and the relatively light framework of the occupant cell, plus interior foams which offer some limited protection, or some vehicle types are now also equipped with supplemental side airbags or curtains which have increased the level of near-side impact protection available to the occupants, but the level of protection does not reach the level which currently exists for frontal crashes.

The results from lateral damage location suggest that strategies and methodologies of occupant protection and injuries reduction are developed from the extent of damage in zones 1, 2 and 3. Two methods are often used to improve vehicle safety in near-side impact crashes.

For peer review only

Method number one is enhancing the structural rigidity of interior panel of side doors to reduce magnitude of door panel crush, such as optimized design of impact bar, foam systems and door interior panels. Method number two is adding the airbags between the occupant and interior panel, i.e. supplemental head curtains and chest airbags which have increased the level of protection available to occupants. Tencer et al. (2005) showed that magnitude of door crush in near-side impact from category 1 (3-8 centimeters of crush) to category 2 (9-15 centimeters of crush) was associated with an average of 0.4 increase in thoracic AIS, a 0.3 increase in abdominal AIS, and a 0.2 increase in pelvis (p<0.0001).[6] In our data, there was not consideration of crush intrusion on MAIS below 3. We analyzed the magnitude of side crush intrusion in near-side impact from zone 2 (about 20-40 centimeters of crush) to zone 3 (about 40-60 centimeters of crush) was associated with an average of 0.22 increase in chest MAIS 3-6, a 0.82 increase in pelvis MAIS 3-6, and a 0.88 increase in upper extremity MAIS 3-6 (p<0.0001).

The results from horizontal damage location suggest that the primary crushed location of impacted vehicle is forward of c-pillar in real world near-side impact crashes, and it is less possibility to impact the rearward of c-pillar for target vehicle. Therefore, for design researches it is useful to maintain the side rigidity of vehicle specially accounted for structure rigidity of side doors, A-pillar, B-pillar and rocker rail.

The results from vertical damage location suggest that the primary crushed location of impacted vehicle is located in upward of rocker rail and downward of windowsill in real world near-side impact crashes, and it is less possibility to impact the upward of windowsill for target vehicle. Considering improving the level of side protection particularly should enhance the rigidity of side doors and rocker rail from perspective of vertical anti-crushed location of vehicle, or consider vehicle front end design, especially for LTV, in motor vehicle safety standards.[15]

In conclusion, over half of crash types in real world are attributed to vehicle-to-vehicle crashes. Rigidity of side doors, A-pillar, B-pillar and rocker rail are priorities for improving the safety level of near-side impact. Because the specific location of damage accounted for MAIS 3-6 injured occupants and 50% injury risk is extent zone 3, zone “Y” and “E” from lateral, horizontal and vertical direction of struck vehicle, respectively. To reduce the risk of deaths and serious injury, protection of head, chest and pelvis should be priorities for injury countermeasure development, because these three body regions accounted for over half of AIS 3-6 injuries suffered from vertical crush zone “E” in PC-LTV near-side impact crashes.

Acknowledgements We would like to thank Dr. Prodeep Mohan (senior scientist of National Crash Analysis Center, George Washington University), Ms. Christina (master graduate student of National Crash Analysis Center, George Washington University), and Mr. Leyu Wang (doctoral graduate student of National Crash Analysis Center, George Washington University) for their very valuable suggestions and software processing to this work.

Contributors YT was the primary investigator, he initiated and designed the study and completed the data processing and manuscript writing. LC and SK participated in developing the study concept and data interpretation. All authors reviewed and approved the final manuscript.

Funding This project was supported by National Crash Analysis Center (NCAC), the George Washington University (GWU) and sponsored by National Natural Science Foundation of China, grant no.51305374 and no.51355001. Open Foundation of State Key Laboratory of Advanced Design and Manufacture for Vehicle Body of Hunan University, grant no. 31215004. Fujian Natural Science Foundation of China, grant no. 2012J05103.

Competing interests None.

REFERENCES

1. **Acierno S**, Kaufman R, Rivara FP, at al. Vehicle mismatch: injury patterns and severity. *Accid Anal and Prev* 2004; **36**: 761-772.
2. **Bradsher K**. The triumph of SUVs. *High and mighty*. 1st Ed. New York: Public Affairs, 2001:382-412
3. **Samaha RR**, Elliott DS. NHTSA side impact research: motivation for upgraded test procedures. Nagoya, Japan, May 2003. *18th Conference on the Enhanced Safety of Vehicles (ESV)* 2003; paper No.492:1-17
4. **Mandell SP**, Mack CD and Bulger EM. Moter vehicle mismatch: a national perspective. *Inj Prev* 2010; **16**: 309-314.

For peer review only

5. **Crashworthiness Data System** (CDS) 2007 coding and editing manual. Washington D.C: Transportation National Highway Traffic Safety Administration, 2007.
6. **Tencer AF**, Kaufman R, Mack C, et al. Factors affecting pelvic and thoracic forces in near side impact crashes: a study of US-NCAP, NASS and CIREN data. *Accid Anal and Prev* 2005; **37**: 287-293.
7. **Insurance Institute for Highway Safety** (IIHS). Putting the crash compatibility issue in perspective. *Insurance Institute for Highway Safety Status Rep*ort 1999; 34:1–11.
8. **Eric MO,** Thomas DK and Barbara M. Will the light truck bumper height-matching standard reduce deaths in cars?. *Traf Inj Prev* 2013; **51**: 292-300.
9. **Society of Automotive Engineering**. *Collision deformation classification of surface vehicle standard-1980*. Warrendale: SAE, 1980.
10. **Charles MF**, Elisa RB and Eric LM. Two-vehicle side impact crashes: the relationship of vehicle and crash characteristics to injury severity. *Accid Anal and Prev* 1997;**29**: 399-406.
11. **Helai H**, Chowdhury S and Mohamed AA. Indexing crash worthiness and crash aggressivity by vehicle type. *Accid Anal and Prev* 2011; **43**: 1364-1370
12. **Toy EL** and Hammitt JK. Safety impacts of suvs, minivans, and pickup trucks in two-vehicle crashes. *Risk Anal* 2003; **23**: 641-650.
13. **Kahane CJ**. Vehicle weight, fatality risk and crash compatibility of model year 1991-99 passenger cars and light trucks. *NHTSA Technical Report* No. DOT HS 809 662, 2003.
14. **Fredette M**, Mambu LS, Chouinard A, at al. Safety impacts due to the incompatibility of suvs, minivans, and pickup trucks in two-vehicle collisions. *Accid Anal and Prev* 2008; 40:1987-1995
15. **Roudsari BS**, Mork CN, Kaufman R, et al. Pedestrian crashes: higher injury severity and mortality rate for light truck vehicles compared with passenger vehicles. *Inj Prev* 2004; **10**:154-158

For peer review only

STROBE Statement—checklist of items that should be included in reports of observational studies

	Item No	Report page
**Title and abstract**	1	Title: Associations between damage location and five main body regions injuries of MAIS 3-6 injured occupants. (Page 1)
		The highest injury risk ratio of MAIS 3-6 injured occupants caused by crush intrusion between 40 centimeters and 60 centimeters in LTV-PC near-side impact collisions, and the damage region of struck vehicle were the zone "E" and "Y". (Page 1)
**Introduction**		
Background/rationale	2	There has been an increase in fatalities resulted from the collision of passenger cars (PC) and light truck vehicles (LTV), because LTV have became the majority of vehicles on the road, resulting in great occurrences of vehicle incompatibility between the body structures of LTV and PC, vehicle mismatch between PC and LTV is associated with deaths and seriously injuries of occupants in automotive crashes. (Page 1, Para.1)
Objectives	3	To estimate the overall maximum Abbreviated Injury Score (MAIS) 3-6 injured occupants of near-side struck vehicle which were associated with specific location of damage in front-to-side impact crashes. (Page 1)
**Methods**		
Study design	4	(a)Cases which only two vehicles were involved in the collision.<br>(b)Cases that resulted in a vehicle rollover were excluded.<br>(c)Crash configuration involved a front-to-side impact.<br>(d)Both of the vehicles involved could be classified as passenger car, and light truck vehicle including mini-van, sport utility vehicle, van, and light pickup truck.<br>(e)Striking vehicle was defined as an impact which resulted in the primary general area of damage being located at the front of the vehicle, also defined as having a direction of force between 3300 and 300 (11'o clock to 1'o clock).<br>(f)Struck vehicle was defined as an impact which resulted in the primary general area of damage being located on either the right or the left side of the vehicle, also defined as having either a direction of force between 300 and 1500 (1'o clock to 5'o clock right side impact), or between 2100 and 3300 (11'o clock to 7'o clock left side impact).<br>(g)Injury analysis was conducted base those injuries sustained by the near-side occupants seated directly adjacent to the struck-side of the vehicle, in a LTV-PC side impact crashes.<br>(Page 2)
Setting	5	MAIS 3-6 injured occupants information was extracted from the NASS/CDS in the year 2007. Struck vehicle collision damage was classified in a three-dimensional system according to J224 Collision Deformation Classification of SAE Surface Vehicle Standard. (Page 1)
Participants	6	Near-side occupants seated directly adjacent to the struck-side of the vehicle with MAIS 3-6 injured, in LTV-PC side impact crashes.(Page 1)
Variables	7	Body regions included head/face/neck, chest, pelvis, upper extremity and lower extremity, and specific location of damage included lateral direction, horizontal direction and vertical direction. (Page 1)
Data sources/ measurement	8*	The extent of lateral damage is classified using a nine-zone extent system relative to specific points on the vehicle structure.<br>Vehicle horizontal areas include zone "P", "F", "B" and "Y", "Z", "D".<br>Vertical location of deformations include "A", "E", "G", "M", or "L". (Page2)
Bias	9	None
Study size	10	The number of crash cases is 4,963 occurred in the year 2007 were selected and after weighted is 2,454,014. (Page2)

For peer review only - http://bmjopen.bmj.com/site/about/guidelines.xhtml

Quantitative variables	11	None
Statistical methods	12	It main used frequency statistic method and confidence interval estimate. (table 3-5)
**Results**		
Participants	13*	(a)The number of MAIS 3-6 injured occupants involved in two passenger vehicle front-to-side crashes.(Page 3,table 2) The number of MAIS 3-6 injured occupants involved lateral location of damage is 564, 705 for horizontal location of damage and vertical location of damage. (table 3-5)
		(b)The missing data of MAIS 3-6 injured occupants involved lateral location of damage is 141, because it was unknown about the AIS of body injury value. (table 3)
		(c) none
Descriptive data	14*	(a)The majority of AIS 3-6 injuries for head/face/neck (36.2%), chest (26.58%) and lower extremity (32.13%) were associated with an extent of damage in zone 4, while pelvis (45.51%), upper extremity (34.41%) were associated with an extent of damage in zone 3. (Page 3, para.4) (b) The highest risk ratio of MAIS 3-6 injuries for head/face/neck, chest, pelvis, upper extremity and lower extremity was respectively about 35.95%, 36.99%, 25.98%, 40% and 47.88% associated with the damage zone "Y" and zone "D".(Page 4, para.2) (c)The majority of all MAIS 3-6 injuries were contribute to the damage location "E", the risk ratio of MAIS 3-6 injuries for head/face/neck, chest, pelvis, upper extremity and lower extremity was about 55.4%, 50.26%, 70.37%, 71.26% and 60.3%, respectively.(Page 5, para.2)
Outcome data	15*	The highest injury risk ratio of MAIS 3-6 injured occupants caused by crush intrusion between 40 centimeters and 60 centimeters in LTV-PC near-side impact collisions, and the damage region of struck vehicle were the zone "E" and "Y". (Page 1)
Main results	16	Table 3-5
Other analyses	17	none
**Discussion**		
Key results	18	The highest injury risk ratio of MAIS 3-6 injured occupants caused by crush intrusion between 40 centimeters and 60 centimeters in LTV-PC near-side impact collisions, and the horizontal location of damage of struck vehicle was zone "E" and the vertical location of damage of struck vehicle was zone "Y". (Page 6, para.4)
Limitations	19	In our data, there was not consideration of crush intrusion on MAIS below 3, and data was retrieved from 2007, was not retrieved from period such as 2004-2013. (Page 6, para.1)
Interpretation	20	Tencer et al. (2005) showed that magnitude of door crush in near-side impact from category 1 (3-8 centimeters of crush) to category 2 (9-15 centimeters of crush) was associated with an average of 0.4 increase in thoracic AIS, a 0.3 increase in abdominal AIS, and a 0.2 increase in pelvis (p<0.0001). (Page 6, para1)
Generalisability	21	Over half of crash types in real world are attributed to vehicle-to-vehicle crashes. Rigidity of side doors, A-pillar, B-pillar and rocker rail are priorities for improving the safety level of near-side impact. (Page 6, para.3)
**Other information**		
Funding	22	Sponsored by National Natural Science Foundation of China, grant no.51305374 and no.51355001. Open Foundation of State Key Laboratory of Advanced Design and Manufacture for Vehicle Body of Hunan University, grant no. 31215004. Fujian Natural Science Foundation of China, grant no. 2012J05103. (Page 6)

For peer review only - http://bmjopen.bmj.com/site/about/guidelines.xhtml
